# COVID-19, Influenza, and RSV in Children and Adults: A Clinical Comparative Study of 12,000 Cases

**DOI:** 10.3390/jcm13061702

**Published:** 2024-03-15

**Authors:** Jae-Hyun Kwon, So-Hyun Paek, Soo-Hyun Park, Min-Jung Kim, Young-Hoon Byun, Ho-Young Song

**Affiliations:** Department of Emergency Medicine, CHA Bundang Medical Center, CHA University School of Medicine, Seongnam 13496, Republic of Korea; hyun21400@naver.com (S.-H.P.); suas11@chamc.co.kr (S.-H.P.); mjtear@naver.com (M.-J.K.); byunyoun84@chamc.co.kr (Y.-H.B.); shyped85@chamc.co.kr (H.-Y.S.)

**Keywords:** COVID-19, influenza, respiratory syncytial viruses, respiratory infections, hospitalization

## Abstract

(1) **Background:** Respiratory virus infections, including Coronavirus disease 2019 (COVID-19), seasonal influenza (FLU), and respiratory syncytial virus (RSV) as prominent examples, can severely affect both children and adults. This study aimed to investigate the clinical characteristics of respiratory viral infections in pediatric and adult populations and to identify determinants influencing patient hospitalization. (2) **Methods:** This retrospective study analyzed the electronic medical records of patients admitted to a regional hospital’s emergency department from 1 January 2015 to 31 December 2022, to investigate the clinical characteristics and hospitalization risk factors associated with these three viruses. (3) **Results:** Infants aged 1 to 11 months were most affected by COVID-19 and RSV, whereas FLU more commonly infected children aged 3 to 5 years. Key factors influencing hospitalization included age and abnormal chest X-ray findings, with higher risks observed in younger children and adults over 65. Notably, the presence of abnormal chest x-ray findings significantly increased the hospitalization risk by 1.9 times [1.5–2.4] in children and 21.4 times [2.4–189.0] in adults. (4) **Conclusions:** This analysis underscores the impact of COVID-19, FLU, and RSV on hospitalization risk, offering insights for managing these respiratory viral infections (RVIs). Age-related risk differences highlight the necessity for tailored strategies, improving understanding of and treatment development for RVIs.

## 1. Introduction

Infections by respiratory viruses, which vary with seasons and periods, significantly influence global health. From pandemics like the Spanish Flu, Severe Acute Respiratory Syndrome coronavirus (SARS-CoV), Middle East Respiratory Syndrome coronavirus (MERS-CoV), and Coronavirus disease 2019 (COVID-19) to more ubiquitous agents such as seasonal influenza (FLU), respiratory syncytial virus (RSV), and parainfluenza, these pathogens are well-recognized contributors to the incidence of acute respiratory infections (ARIs) that necessitate hospital admissions [1,2,3].

The emergence in 2019 of the novel coronavirus, COVID-19, led to an increase in mortality and hospitalization rates across all age groups due to the infection [4,5]. Conversely, during this period, social measures such as social distancing and wearing masks contributed to a decrease in emergency department (ED) visits [5]. Additionally, the incidence of infections from respiratory viruses other than COVID-19 displayed varying trends, with noticeable decreases [6,7] or increases [8] in different countries.

Recurring annually, FLU is a significant viral disease leading to severe cases and fatalities. It causes a high fever, chills, and respiratory symptoms in both adults and children, and is known for its high mortality rate during each seasonal outbreak [9,10].

RSV is a common respiratory virus in infants, often presenting mild cold-like symptoms in adults but potentially causing severe bronchiolitis in children, increasing hospitalization and mortality rates in younger age groups [3,11,12]. Since the 1970s, research has intensified around RSV infections in the elderly, indicating higher risks and potential severe cardiac and pulmonary complications [13,14,15]. With the global increase in life expectancy, RSV demands further research due to its significant impact on vulnerable populations.

Investigations into the severity of infections caused by COVID-19, FLU, and RSV have predominantly concentrated on pediatric and adult populations independently, often scrutinizing the effects of either individual viruses or their combined impact [2,4,6,7,8,10,11,13,16,17,18,19]. Notwithstanding, comparative analyses delineating the clinical manifestations of these pathogens among individuals younger than 18 versus those of adults remain markedly limited.

This study endeavors to examine the clinical characteristics of respiratory viral infections within both pediatric and adult cohorts, in addition to identifying determinants influencing the likelihood of hospitalization. Such comparative research is imperative for elucidating age-associated discrepancies and pinpointing factors that could facilitate the prompt identification of patients necessitating medical intervention, thereby enhancing our comprehension of these infections and optimizing patient care strategies.

## 2. Materials and Methods

### 2.1. Study Design and Setting

In this investigation, we retrospectively analyzed electronic medical records (EMRs) of patients who were discharged from the ED with diagnoses of COVID-19, seasonal influenza, or RSV, covering the period from 1 January 2015 to 31 December 2022. The study was conducted at a tertiary hospital in Gyeonggi Province, South Korea, which annually serves approximately 25,000 pediatric patients under 18 and around 55,000 adults over 18.

The Institutional Review Board (CHAMC 2023-07-026) approved the study, with a waiver for obtaining informed consent from the participants.

### 2.2. Selection of Participants

All patients admitted to the ED during the specified period were identified using the ICD-10 codes for COVID-19, seasonal influenza, and RSV from the 10th revision. Patients with diagnostic codes related to these viruses were included and subsequently divided into two groups: pediatric patients under 18, and adults aged 18 and over.

### 2.3. Data Collection

**Figure 1:**
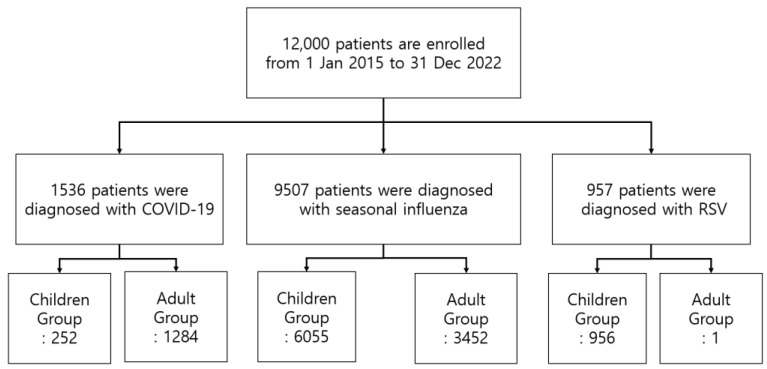
Patient flow chart.

This stage involved reviewing EMRs from the hospital for each participant, gathering data such as age (in months) and gender, and categorizing patients into detailed subgroups. For pediatric patients under 18, classifications included neonate (0 months), infant (1–11 months), toddler (1–2 years), preschooler (3–5 years), school age (6–12 years), and adolescent (13–17 years). Adult patients were divided into adult (18–65 years) and elderly (over 65 years). The study analyzed emergency department length of stay (EDLOS), discharge outcome, initial vital signs, blood tests, and imaging findings from chest X-rays and CT scans. Imaging reports, interpreted by experienced radiologists, were used to define abnormal findings based on specific diagnoses. Patients were grouped into COVID-19, FLU, and RSV infection groups, with a subdivision into pediatric and adult categories (Figure 1). Patients with multiple infections were excluded. A sub-analysis explored clinical differences between children and adults within each virus group, identifying risk factors influencing hospitalization outcomes.

### 2.4. Data Analysis

Data analysis was performed using SPSS statistical software (SPSS for Windows, ver.29.0; IBM SPSS Statistics, Armonk, NY, USA). Descriptive statistics were presented as either means ± standard deviations or medians (interquartile ranges (IQR)), with values rounded to two decimal places. Categorical variables were described by frequency and percentage. Statistical analyses employed Pearson’s chi-square test and Fisher’s exact test, with multiple regression analysis to identify risk factors for hospitalization in pediatric and adult patient groups. A *p*-value of less than 0.05 was considered statistically significant.

## 3. Results

From 1 January 2015 to 31 December 2022, there were 12,000 patients diagnosed with either COVID-19, FLU, or RSV. Specifically, 1536 had COVID-19, with 252 pediatric patients under 18 and 1284 adults. FLU accounted for 9507 cases, split between 6055 children and 3452 adults. RSV diagnoses included 956 children and 1 adult, totaling 957. There were no cases of dual infections among these three viruses (Figure 1).

### 3.1. Baseline Characteristics

Table 1 shows clinical differences among the RVI groups, with COVID-19 patients having the highest average age at 49.2 ± 27.7 years, FLU at 19.5 ± 23.0 years, and RSV at 0.6 ± 2.8 years, indicating the youngest average age in RSV patients. The highest proportion was in the adult group aged 18 to 64, at 29.6%. EDLOS averaged 239.4 ± 554.2 min, with COVID-19 patients having the longest stays. Discharge rates were highest overall at 79.3%, with 88.3% of the FLU group going home, and the highest hospitalization rate was in the RSV group. Imaging abnormalities were most frequent in the FLU group, at 91.9% for chest X-rays and 95.0% for chest CT.

### 3.2. Comparison of Children and Adult Groups of COVID-19/FLU/RSV-Infected Patients

Table 2 divides the 1536 patients diagnosed with COVID-19 into pediatric (252) and adult (1284) groups, highlighting a higher number of boys in the pediatric group and a higher proportion of women in the adult group. The average age was 3.7 ± 5.1 years in children and 58.1 ± 20.7 years in adults, with infants 1–11 months old being predominant in the pediatric group. Both groups showed a similar EDLOS, but a higher discharge rate was noted in adults compared to a 48.4% hospitalization rate in children. There were no significant differences in WBC, serum lactate, procalcitonin levels, or the presence of lesions in chest x-rays between the groups.

Table 3 discusses the 9507 patients diagnosed with FLU, with 6055 children and 3452 adults. Boys were more prevalent in the pediatric group, while women were the majority in the adult group. Children aged 3–5 years were most common, with adults mostly between 18–64 years. Adults had a longer average EDLOS, with discharge being the most common outcome for both groups. Pediatric patients had a higher rate of chest x-ray abnormalities at 21.5%.

Table 4 shows the analysis of 957 RSV-infected patients, with 956 children and 1 adult diagnosed. The majority in the pediatric group were boys, making up 55.5%, and infants constituted 57.8%. A significant portion—62.8%, or 600 patients—were hospitalized. All imaging studies conducted in the pediatric RSV group showed abnormal findings.

### 3.3. Odds Ratio of Hospitalization for Children and Adult Groups

**Figure 2:**
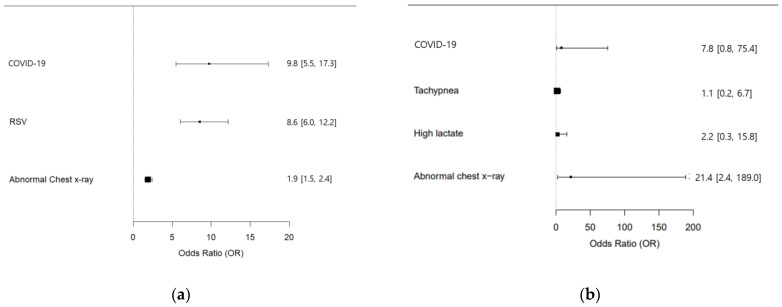
Forest plot of odds ratios for hospitalized children and adult groups. (**a**) Adjusted odds ratio of children group. (**b**) Adjusted odds ratio of adult group.

To identify factors affecting hospital admissions, odds ratios were calculated using univariate analysis with age and disease as variables. Neonates (0 months) in the pediatric group had the highest admission risk at 80.0 times, with infants (1–11 months) at 8.9 times, and toddlers (1–2 years) at 2.2 times. Adults over 65 had a 3.7 times higher admission risk compared to those aged 18–64. Pediatric admissions for COVID-19 were 9.7 times and RSV 8.0 times higher than for influenza. In adults, COVID-19 risk was 0.9 times higher than that of influenza, which is not significant. Multivariate analysis indicated pediatric admissions for COVID-19 were 9.8 times and RSV 8.6 times higher than for influenza, with rates of chest x-ray abnormalities in COVID-19 patients 1.9 times higher than in influenza patients. Adults showed a 9.8 times higher risk of COVID-19, and an increase of 21.4 times for abnormal chest x-rays (Table 5 and Table 6) (Figure 2).

## 4. Discussion

This research, conducted over approximately 8 years from January 2015 to 2022, involved 12,000 patients in a single hospital, examining clinical differences between pediatric and adult groups for three major respiratory viruses: COVID-19, FLU, and RSV. It also sought to identify factors affecting hospital admissions in both children and adults, emphasizing the prevalence of lower RVIs in infants and their role as a common cause of pediatric pneumonia and hospitalization [16,20]. The pandemic declaration of COVID-19 in 2020 led to a significant increase in research focused on this virus, alongside traditional pathogens like FLU and RSV.

In a study comparing mortality rates among FLU, RSV, rhinovirus, metapneumovirus, and COVID-19 infections, COVID-19 initially showed the highest mortality. Over time, the mortality rate differences between COVID-19 and other viruses lessened [4]. This research also found a higher mortality rate among COVID-19 patients compared to those with FLU or RSV, mainly among adults. Among the 252 pediatric COVID-19 patients, despite a higher hospitalization rate compared to adults, there were no fatalities, aligning with studies suggesting children manage COVID-19 infections more effectively [21]. Extended EDLOS for COVID-19 patients were likely due to isolation requirements until hospital isolation beds became available. FLU has been a significant source of morbidity and mortality [3,22,23], with studies in the U.S. showing that mortality rates are highest for influenza A(H3N2), followed by RSV, influenza B, and A(H1N1), often linked to underlying respiratory and circulatory conditions [9]. FLU mortality increases with age, particularly posing a higher risk for those over 65 [9].

RSV is a leading cause of hospital admissions among infants under 2 years old in the U.S. and Germany [8,16,17]. During the three years following the 2020 COVID-19 pandemic’s onset, hospitalizations due to RSV in infants under 1 year in Germany exceeded half of all cases, with higher rates in 1–2-year-olds post-pandemic than pre-pandemic [8]. This indicates RSV’s significant impact on pediatric respiratory illnesses, independent of COVID-19. This study found a high risk of hospitalization due to RSV in children, especially in younger age groups, aligning with previous findings.

RSV is recognized for causing significant infections not only in children, but also in the elderly [3,17,19,24,25]. Recent reports suggest that RSV in hospitalized adults can lead to severe respiratory illnesses akin to those from FLU complications [18], with studies indicating that 78% of deaths in individuals over 65 are related to RSV [9]. Additionally, viral infections constitute about 22.5% of severe hospital-acquired pneumonia cases, with RSV and parainfluenza being the most frequently detected [26], suggesting that viral infections, including RSV, can exacerbate the severity and mortality of respiratory diseases in adults as well as children.

Respiratory viruses like RSV have been extensively studied, but understanding their impact across different age groups, especially between children and adults, remains crucial. In this study, a total of 957 patients were diagnosed with RSV, and a very high percentage, 62.7%, required hospitalization. Among the adults, there was only one diagnosed case, which was a limitation due to the relatively small number of cases. However, the significance lies in the fact that the patient was of advanced age, at 84 years; had no other concurrent viral infections; and, despite a hopeless discharge, hospitalization was necessary. Korea is now beginning to experience population aging, and as the proportion of elderly people increases, the risk of RSV infections, which have been predominantly seen in children, is gradually rising. Therefore, research on this subject is deemed essential. Additionally, investigating the respiratory effects of viruses such as parainfluenza and bocavirus across age groups is essential for developing health strategies, including vaccination policies and age-appropriate diagnostic and hospitalization protocols. Additionally, future research on respiratory virus infections and the development of age-appropriate diagnostic and hospitalization strategies will be necessary.

This study has several limitations. First, it was conducted during the period from 2020 to 2022, which coincided with the global spread of COVID-19. During this time, various preventive measures such as social distancing and mask-wearing were implemented, which may have led to a reduction in the incidence of other viruses. This could introduce selection bias or confounding variables.

Second, the study focused solely on viral infections and did not consider other diagnoses such as bacterial pneumonia or bronchitis. It is possible that factors such as bacterial infections may have also influenced hospitalization rates or mortality. The literature indicates that bacterial co-infections can significantly influence the outcomes of patients with viral respiratory infections, including hospitalization rates and mortality. In a study examining acute respiratory infections in China, it was found that a notable percentage of patients tested positive for both viral and bacterial pathogens, with co-infections affecting a considerable proportion of these cases, particularly among children [27]. Another study specifically investigated bacterial co-infections in patients with community-acquired pneumonia caused by SARS-CoV-2, influenza, and respiratory syncytial virus (RSV), revealing that bacterial co-infections at admission were less frequent in SARS-CoV-2 patients compared to those with influenza or RSV. However, the presence of bacterial co-infection was associated with increased inflammatory markers, underscoring the impact of such co-infections on patient outcomes [28].

These findings underscore the importance of considering bacterial co-infections during the management and treatment of viral respiratory infections, as they can complicate the clinical course and affect the prognoses of patients. Identifying and addressing these co-infections early can potentially improve patient outcomes and reduce the risk of severe complications, including the need for hospitalization and the risk of mortality.

Last, the study was conducted in a single comprehensive hospital, which may introduce selection bias. Further research and validation from diverse settings are needed to support these findings.

Over the past centuries, humanity has battled respiratory viruses, and it is believed that conducting research tailored to specific types of respiratory viruses and responding appropriately can lead to finding ways to efficiently utilize limited medical resources.

## 5. Conclusions

This study aimed to identify clinical differences between children and adults infected with three representative respiratory viruses and to determine the factors influencing hospitalization. Through this research, it was found that in the case of children, the risk of hospitalization increased with younger age. Factors such as COVID-19 infection and RSV infection were analyzed as factors influencing hospitalization, compared to seasonal influenza. Similarly, in adults, it was observed that the risk of hospitalization was higher in adults aged 65 and older. Therefore, when dealing with respiratory virus infections, it is advisable to reassess the likelihood of hospitalization based on age for both children and adults and make decisions regarding hospitalization accordingly.

## Figures and Tables

**Table 1:** Demographics of COVID-19/FLU/RSV-infected patients.

	Total	COVID-19	FLU	RSV	*p*-Value
	*N* = 12,000 (%)	*N* = 1536 (%)	*N* = 9507 (%)	*N* = 957 (%)	
Age (mean ± SD), year	21.8 ± 25.6	49.2 ± 27.7	19.5 ± 23.0	0.6 ± 2.8	<0.001
Male sex, *N* (%)	5766 (48.1)	757 (49.3)	4478 (47.1)	531 (55.5)	<0.001
Patient age groups, *N* (%)					
Neonate (0 months)	90 (0.8)	12 (0.8)	1 (0)	77 (8.0)	<0.001
Infant (1–11 months)	1174 (9.8)	93 (6.1)	528 (5.6)	553 (57.8)	<0.001
Toddler (1–2 years)	2033 (16.9)	54 (3.5)	1676 (17.6)	303 (31.7)	<0.001
Preschooler (3–5 years)	2031 (16.9)	27 (1.8)	1983 (20.9)	21 (2.2)	<0.001
School Age (6–12 years)	1598 (13.3)	40 (2.6)	1556 (16.4)	2 (0.2)	<0.001
Adolescent (13–17 years)	337 (2.8)	26 (1.7)	311 (3.3)	-	<0.001
Adult (18–64 years)	3547 (29.6)	718 (46.7)	2829 (29.8)	-	<0.001
Elderly (>65 years)	1190 (9.9)	566 (36.8)	623 (6.6)	1 (0.1)	<0.001
EDLOS	12,000 (100)				<0.001
mean ± SD, (min)	239.4 ± 554.2	629.4 ± 1126.2	174.0 ± 383.8	263.8 ± 257.2	
median (IQR) (min)	112.5 (126.8)	286.0 (421.0)	96.0 (89.0)	190.0 (153.5)	
Disposition, No. (%)	12,000 (100)	1536 (12.8)	9507 (79.2)	957 (8.0)	<0.001
Discharge	9513 (79.3)	767 (49.9)	8393 (88.3)	353 (36.9)	
Admission	2028 (16.9)	534 (34.8)	894 (9.4)	600 (62.7)	
Transfer	145 (1.2)	137 (8.9)	7 (0.1)	1 (0.1)	
Hopeless discharge	259 (2.2)	44 (2.9)	212 (2.2)	3 (0.3)	
Death	19 (0.2)	18 (1.2)	1(0)	-	
Other	36 (0.3)	36 (2.3)	-	-	
Initial ED vital signs					
SBP (mean ± SD), mmHg	126.8 ± 20.0	131.5 ± 23.6	125.5 ± 18.6	109.3 ± 15.6	<0.001
DBP (mean ± SD), mmHg	74.5 (12.2)	75.3 (14.3)	74.3 (11.5)	69.2 (12.6)	0.005
HR (mean ± SD), beats per min	123.6 (30.1)	103.4 (29.8)	123.5 (27.8)	157.9 (19.1)	<0.001
RR (mean ± SD), breaths per min	23.8 (7.3)	21.2 (6.1)	22.7 (4.8)	39.8 (10.9)	<0.001
SpO2 (mean ± SD), (%)	98.4 (3.0)	97.4 (4.3)	98.7 (2.7)	98.0 (2.8)	<0.001
BT (mean ± SD), (°C)	38.2 (1.0)	37.6 (1.0)	38.4 (0.9)	37.6 (0.9)	<0.001
Laboratory findings					
WBC, 10^3^/L	7.9 (3.9)	7.7 (4.6)	7.5 (3.5)	10.5 (3.8)	<0.001
Serum lactate, mmol/L	2.2 (1.7)	2.5 (2.9)	2.0 (1.0)	2.5 (0.8)	<0.001
Procalcitonin, ng/mL	1.1 (6.3)	1.1 (6.5)	1.0 (5.7)	1.0 (7.0)	0.984
Abnormal images *					
Chest X-ray	1285 (17.8)	29 (2.3)	1181 (91.9)	75 (5.8)	<0.001
Chest CT	40 (95.2)	2 (5.0)	38 (95.0)	-	<0.001

COVID-19, coronavirus disease 2019; FLU, seasonal influenza; RSV, respiratory syncytial virus; SD, standard deviation; EDLOS, emergency department length of stay; IQR, interquartile range; ED, emergency department; SBP, systolic blood pressure; DBP, diastolic blood pressure; HR, heart rate; RR, respiratory rate; SpO2, saturation pulse oximeter; BT, body temperature; WBC, whole blood count; CT, computed tomography. * Abnormal images: this includes any noted irregularities in chest X-rays and chest CT, such as lower respiratory infection, bronchitis, pleural effusion, or pneumonia.

**Table 2:** Comparison of children and adult groups of COVID-19-infected patients.

	Total = 1536	Children Group	Adult Group	*p*-Value
		*N* (%)	*N* (%)	
Patient sex	1536 (100)	252 (100)	1284 (100)	
Male sex, *N* (%)	757 (49.3)	153 (60.7)	604 (47.0)	<0.001
Patient age groups, *N* (%)				
Mean ± SD (year)	49.2 ± 27.7	3.7 ± 5.1	58.1 ± 20.7	<0.001
Neonate (0 months)	12 (0.8)	12 (4.8)		
Infant (1–11 months)	93 (6.1)	93 (36.9)		
Toddler (1–2 years)	54 (3.5)	54 (21.4)		
Preschooler (3–5 years)	27 (1.8)	27 (10.7)		
School Age (6–12 years)	40 (2.6)	40 (15.9)		
Adolescent (13–17 years)	26 (1.7)	26 (10.3)		
Adult (18–64 years)	718 (46.7)		718 (55.9)	
Elderly (>65 years)	566 (36.8)		566 (44.1)	
EDLOS				
Mean ± SD, (min)	629.4 ± 1126.2	587.9 ± 863.7	637.6 ± 1171.0	0.522
Median (IQR) (min)	286.0 (421.0)			
Disposition (%)	1536 (12.8)	252 (16.4)	1284 (83.6)	<0.001
Discharge	767 (49.9)	85 (33.7)	682 (53.1)	
Admission	534 (34.8)	122 (48.4)	412 (32.1)	
Transfer	137 (8.9)	18 (7.1)	119 (9.3)	
Hopeless discharge	44 (2.9)	1 (0.4)	43 (3.3)	
Death	18 (1.2)	0 (0)	18 (1.4)	
Other	36 (2.3)	26 (10.3)	10 (0.8)	
Initial ED vital signs				
SBP (mean ± SD), mmHg	131.5 ± 23.6	112.0 ± 14.1	133.2 ± 23.5	<0.001
DBP (mean ± SD), mmHg	75.3 ± 14.3	68.8 ± 12.2	75.9 ± 14.3	<0.001
HR (mean ± SD), beats per min	103.4 ± 29.8	150.4 ± 29.2	94.2 ± 19.3	<0.001
RR (mean ± SD), breaths per min	21.2 ± 6.1	31.1 ± 9.3	19.4 ± 2.9	<0.001
SpO2 (mean ± SD), (%)	97.4 ± 4.3	99.0 ± 2.4	97.1 ± 4.5	<0.001
BT (mean ± SD), (°C)	37.6 ± 1.0	38.2 ± 0.9	37.5 ± 1.0	<0.001
Laboratory findings				
WBC, 10^3^/L	7.7 (4.6)	8.3 (4.5)	7.6 (4.7)	0.065
Serum lactate, mmol/L	2.5 (2.9)	2.4 (1.2)	2.5 (3.1)	0.861
Procalcitonin, ng/mL	1.1 (6.5)	0.2 (0.3)	1.2 (6.9)	0.137
Abnormal images (%)				
Chest X-ray	29 (2.3)	9 (17.0)	20 (26.7)	0.20
Chest CT	2 (5.0)		2 (66.7)	

COVID-19, coronavirus disease 2019; SD, standard deviation; EDLOS, emergency department length of stay; IQR, interquartile range; ED, emergency department; SBP, systolic blood pressure; DBP, diastolic blood pressure; HR, heart rate; RR, respiratory rate; SpO2, saturation pulse oximeter; BT, body temperature; WBC, whole blood count; CT, computed tomography. Abnormal images: this includes any noted irregularities in chest X-rays and chest CT, such as lower respiratory infection, bronchitis, pleural effusion, or pneumonia.

**Table 3:** Comparison of children and adult groups of FLU-infected patients.

	Total = 9507	Children Group	Adult Group	*p*-Value
		*N* (%)	*N* (%)	
Patient sex	9507 (100)	6055 (100)	3452 (100)	
Male sex, *N* (%)	4478 (47.1)	3232 (53.4)	1246 (36.1)	<0.001
Patient age groups, *N* (%)				
Mean ± SD (year)	19.5 (23.0)	4.5 (3.8)	45.8 (18.5)	<0.001
Neonate (0 months)	1 (0)	1 (0.0)		
Infant (1–11 months)	528 (5.6)	528 (8.7)		
Toddler (1–2 years)	1676 (17.6)	1676 (27.7)		
Preschooler (3–5 years)	1983 (20.9)	1983 (32.7)		
School Age (6–12 years)	1556 (16.4)	1556 (25.7)		
Adolescent (13–17 years)	311 (3.3)	311 (5.1)		
Adult (18–64 years)	2829 (29.8)		2829 (82.0)	
Elderly (>65 years)	623 (6.6)		623 (18.0)	
EDLOS				
Mean ± SD, (min)	174.0 ± 383.8	125.0 ± 156.8	259.7 ± 592.4	<0.001
Disposition (%)	9507 (100)	6055 (100)	3452 (100)	<0.001
Discharge	8393 (88.3)	5531 (91.3)	2862 (82.9)	
Admission	894 (9.4)	495 (8.2)	399 (11.6)	
Transfer	7 (0.1)	3 (0.0)	4 (0.1)	
Hopeless discharge	212 (2.2)	26 (0.4)	186 (5.4)	
Death	1(0)	0 (0)	1 (0)	
Other	-	-	-	
Initial ED vital signs				
SBP (mean ± SD), mmHg	125.5 ± 18.6	113.6 ± 12.3	130.1 ± 18.6	<0.001
DBP (mean ± SD), mmHg	74.3 ± 11.5	69.1 ± 9.3	76.3 ± 11.7	<0.001
HR (mean ± SD), beats per min	123.5 ± 27.8	138.2 ± 23.5	100.4 ± 16.1	<0.001
RR (mean ± SD), breaths per min	22.7 ± 4.8	25.1 ± 4.8	19.3 ± 2.0	<0.001
SpO2 (mean ± SD), (%)	98.7 ± 2.7	99.0 ± 2.1	97.9 ± 3.6	<0.001
BT (mean ± SD), (°C)	38.4 ± 0.9	38.5 ± 0.9	38.0 ± 0.9	<0.001
Laboratory findings				
WBC, 10^3^/L	7.5 (3.5)	8.4 (3.9)	7.0 (3.1)	<0.001
Serum lactate, mmol/L	2.0 (1.0)	2.1 (0.9)	1.8 (1.4)	<0.001
Procalcitonin, ng/mL	1.0 (5.7)	0.3 (0.9)	1.7 (7.7)	0.003
Abnormal images (%)				
Chest X-ray	1181 (91.9)	963 (21.5)	218 (8.8)	<0.001
Chest CT	38 (95.0)	-	38 (97.4)	-

FLU, seasonal influenza; SD, standard deviation; EDLOS, emergency department length of stay; IQR, interquartile range; ED, emergency department; SBP, systolic blood pressure; DBP, diastolic blood pressure; HR, heart rate; RR, respiratory rate; SpO2, saturation pulse oximeter; BT, body temperature; WBC, whole blood count; CT, computed tomography. Abnormal images: this includes any noted irregularities in chest X-rays and chest CT, such as lower respiratory infection, bronchitis, pleural effusion, or pneumonia.

**Table 4:** Comparison of children and adult groups of RSV-infected patients.

	Total = 957	Children Group	Adult Group	*p*-Value
		*N* (%)	*N* (%)	
Patient sex	957 (8.0)	956 (100)	1 (100)	
Male sex, *N* (%)	531 (55.5)	531 (55.5)	-	0.445
Patient age groups, *N* (%)				0.001
Mean ± SD (year)	0.6 ± 2.8	0.5 ± 0.8	84.0 (-)	<0.001
Neonate (0 months)	77 (8.0)	77 (8.1)		
Infant (1–11 months)	553 (57.8)	553 (57.8)		
Toddler (1–2 years)	303 (31.7)	303 (31.7)		
Preschooler (3–5 years)	21 (2.2)	21 (2.2)		
School Age (6–12 years)	2 (0.2)	2 (0.2)		
Adolescent (13–17 years)	-			
Adult (18–64 years)	-			
Elderly (>65 years)	1 (0.1)		1 (100)	
EDLOS				
Mean ± SD, (min)	263.8 ± 257.2	261.8 ± 249.6	2205.0 (-)	<0.001
Disposition (%)	957 (8.0)	956 (99.9)	1 (0.1)	0.004
Discharge	353 (36.9)	353 (36.9)	-	
Admission	600 (62.7)	600 (62.8)	-	
Transfer	1 (0.1)	1 (0.1)	-	
Hopeless discharge	3 (0.3)	2 (0.2)	1 (100)	
Death	-	-	-	
Other	-	-	-	
Initial ED vital signs				
SBP (mean ± SD), mmHg	109.3 ± 15.6	107.3 ± 13.7	140	0.037
DBP (mean ± SD), mmHg	69.2 ± 12.6	68.3 ± 12.5	82.0	0.309
HR (mean ± SD), beats per min	157.9 ± 19.1	158.0 ± 18.9	71.0	<0.001
RR (mean ± SD), breaths per min	39.8 ± 10.9	39.8 ± 10.9	26.0	0.207
SpO2 (mean ± SD), (%)	98.0 ± 2.8	98.0 ± 2.8	98.0	0.986
BT (mean ± SD), (°C)	37.6 ± 0.9	37.6 ± 0.9	37.7	0.935
Laboratory findings				
WBC, 10^3^/L	10.5 (3.8)	10.5 (3.8)	10.4	0.972
Serum lactate, mmol/L	2.5 (0.8)	2.5 (0.8)	1.7	0.379
Procalcitonin, ng/mL	1.0 (7.0)	1.0 (7.0)	0.5	0.936
Abnormal images (%)				
Chest X-ray	75 (5.8)	75 (100)	-	
Chest CT	-	-	-	

RSV, respiratory syncytial virus; SD, standard deviation; EDLOS, emergency department length of stay; IQR, interquartile range; ED, emergency department; SBP, systolic blood pressure; DBP, diastolic blood pressure; HR, heart rate; RR, respiratory rate; SpO2, saturation pulse oximeter; BT, body temperature; WBC, whole blood count; CT, computed tomography. Abnormal images: this includes any noted irregularities in chest X-rays and chest CT, such as lower respiratory infection, bronchitis, pleural effusion, or pneumonia.

**Table 5:** Odds ratio of hospitalization for adult and children groups (univariate analysis).

Children Group				Adult Group			
	*N*	OR (95% CI)	*p*-Value		*N*	OR (95% CI)	*p*-Value
Neonate (0 months)	86	80.0 (25.6–250.3)	<0.001	Adult (18–64 year)	885	1.0 †	-
Infant (1–11 months)	631	8.9 (5.2–15.2)	<0.001	Elderly (>65 year)	651	3.7 (3.2–4.3)	<0.001
Toddler (1–2 years)	247	2.2 (1.3–3.8)	0.003				
Preschooler (3–5 years)	135	1.9 (1.1–3.3)	0.21				
School Age (6–12 years)	123	2.1 (1.2–3.6)	0.01				
Adolescent (13–17 years)	17	1.0 †	_				
FLU	498	1.0 †	-	FLU	1056	1.0 †	-
COVID-19	12	9.7 (7.3–13.0)	<0.001	COVID-19	480	0.9 (0.8–1.1)	0.5
RSV	601	8.0 (6.6–9.7)	<0.001				

OR, odds ratio; CI, confidence interval; FLU, seasonal influenza; COVID-19, coronavirus disease 2019; RSV, respiratory syncytial virus. † This group served as the reference group.

**Table 6:** Adjusted odds ratio of hospitalization for adult and children groups (multivariable analysis).

Children Group				Adult Group			
	*N*	AOR (95% CI)	*p*-Value		*N*	AOR (95% CI)	*p*-Value
FLU	1056	1.0 †	-	FLU	498	1.0 †	-
COVID-19	480	9.8 (5.5, 17.3)	<0.001	COVID-19	12	7.8 (0.8, 75.4)	0.073
RSV	610	8.6 (6.0, 12.2)	<0.001	RSV	1	-	-
				Tachypnea ††		1.1 (0.2–6.7)	0.957
				High lactate ‡		2.2 (0.3–15.8)	0.417
Abnormal chest X-ray		1.9 (1.5, 2.4)	<0.001	Abnormal chest X-ray		21.4 (2.4–189.0)	0.006

AOR, adjusted odds ratio; CI, confidence interval; FLU, seasonal influenza; COVID-19, coronavirus disease 2019; RSV, respiratory syncytial virus. † This group served as the reference group. †† Tachypnea: over 20 breaths per minute. ‡ High lactate: over 2 mmol/L. Abnormal chest X-ray: This includes any noted irregularities in chest X-rays, such as lower respiratory infection, bronchitis, pleural effusion, or pneumonia.

## Data Availability

The data presented in this study are available on request from the corresponding author. The data are not publicly available due to restrictions imposed by hospital review, which mandates the destruction of the data within three years.
